# Insights into the personality of 193 German elite youth football players and potential implications for the development of motor performance and injury risk

**DOI:** 10.3389/fspor.2026.1713872

**Published:** 2026-02-16

**Authors:** Sebastian Viktor Waldemar Schulz, Julia Holzapfel, Lynn Matits, Eric Schwarz, Daniel Alexander Bizjak, Achim Jerg, Markus Kiefer, Johannes Kirsten, Alexander-Stephan Henze

**Affiliations:** 1 Sports and Rehabilitation Medicine, University Hospital Ulm, Ulm, Germany; 2 Department of Psychiatry, Section for Cognitive Electrophysiology, Ulm University, Ulm, Germany; 3 Clinical & Biological Psychology, Institute of Psychology and Education, Ulm University, Ulm, Germany

**Keywords:** athletes, athletic injuries, character, performance tests, personality inventory, soccer, temperament

## Abstract

**Introduction:**

Sport-specific performance in football is influenced not only by physical, technical, and tactical factors but also by psychological characteristics such as personality. Previous research suggests traits like extraversion and conscientiousness are positively associated with athletic outcomes. A deeper understanding of personality may allow for more individualized training and support, potentially enhancing both short-term performance and long-term development. The present study aimed firstly to describe the prevalence and distribution of personality dimensions according to Cloninger's biopsychosocial model, assessed with the Junior Temperament and Character Inventory (JTCI), in German elite youth football players, and secondly to explore potential associations of these dimensions with motor performance and injury risk.

**Methods:**

A total of 193 players (aged 11–18 years, mean 14.6 ± 1.9) from two elite academies were assessed during the 2023/2024 pre-season. In addition to the JTCI, body composition, 30 m sprint, heading jump, and injury history were collected. Analyses included Spearman correlations, multiple linear regression and moderation analyses (with HC3 robust standard errors), as well as logistic regression for injury occurrence.

**Results:**

The personality dimensions of Persistence (mode: 95%–100%) and Self-Directedness (mode: 85%–90%) tended to be distributed toward high percentile ranks, while Novelty Seeking (mode: 20%–25%) and Harm Avoidance (mode: 10%–15%) tended toward low ranks. Age consistently predicted sprint performance and injury frequency, while no personality dimension showed significant associations with sprinting, jumping, or injury outcomes.

**Discussion:**

To our knowledge, this is the first study applying Cloninger's model in German elite youth football. These findings provide rare descriptive insights into adolescent athletes' personality profiles and suggest that personality may play a greater role in long-term development, resilience, and talent progression than in immediate physical performance.

## Introduction

The performance of football players is influenced by a complex interplay of technical, physical, tactical, and psychological skills (1–3). In recent years, the role of psychological and particularly personality-related factors has gained increasing attention in youth talent development programs (2, 4–8). In Germany football is one of the most popular sports (9, 10), and the German Football Association (Deutscher Fußballbund, DFB) supports youth talent development by 58 elite academies, aiming to prepare players for professional competition (11).

In the scientific debate on personality and sporting performance, three central perspectives have been discussed. The first perspective is the debate between those who are credulous and those who are skeptical. The former assumes that personality traits reliably predict performance, while the latter emphasizes weak or inconsistent associations (12, 13). The second perspective is the development vs. selection hypothesis, which addresses whether sports participation actively shapes certain personality profiles through training and socialization processes, or whether athletes with specific profiles are more likely to be selected and retained in talent pathways (14, 15). The third perspective is the performance hypothesis, which assumes that specific traits such as high conscientiousness, emotional stability and self-regulation directly contribute to improved performance, resilience and reduced injury risk (14, 16). Recent systematic reviews and meta-analyses have highlighted meaningful, yet modest, links between personality and sporting performance (14, 16), while also identifying important moderating factors, such as age, sport type and level of competition. Prospective work shows that competitive anxiety and psychological stress predict injury occurrence in adolescent soccer players (17, 18). In addition, a recent consensus statement highlights stress, anxiety, and inadequate coping as central psychological risk mechanisms in youth sport (19), aligning well with the patterns observed in the present study. Together, these perspectives emphasize the importance of considering personality in youth elite football, while acknowledging that its predictive value for short-term outcomes is still being debated.

Several models have been proposed to conceptualize personality, including the Five-Factor Model (20), the Affective Neuroscience Personality Scales [ANPS (21)], and the Cognitive-Affective Personality System [CAPS (22)]. The present study focuses on Cloninger's biosocial model (23), which explains how personality differs among individuals across developmental stages, from childhood through adolescence to adulthood. The model distinguishes four temperament dimensions (Novelty Seeking, Harm Avoidance, Reward Dependence, and Persistence) and three-character dimensions (Self-Directedness, Cooperativeness, and Self-Transcendence), providing a comprehensive framework for understanding personality development in healthy and clinical populations (for a detailed overview of all dimensions and their psychological relevance, see Supplementary Table S1). Cloninger's model capture biologically rooted tendencies and socially shaped aspects of self-regulation relevant for adolescent development.

Personality theories also offer behavioral mechanisms that help explain how traits may relate to injury risk. In the Five-Factor Model, low conscientiousness and high neuroticism have been linked to reduced self-regulation, heightened stress reactivity, and risk-taking behavior—factors consistently associated with increased injury vulnerability in youth and adult athletes (13, 16, 24, 25). Within Cloninger's framework, high Novelty Seeking and low Self-Directedness are related to impulsive decision-making, emotional instability, and poorer adherence to training or recovery routines (23, 26), providing a plausible pathway through which personality traits may indirectly influence injury susceptibility in youth football players.

Cloninger and colleagues (23) describe personality development as a dynamic process in which temperament remains relatively stable, while character dimensions evolve in response to biological and social experiences (27). The temperament refers to automatic reaction tendencies to stimuli, which form the biological basis of personality. The character is more strongly formed by socio-cultural learning processes and reflects the personal goals and values (28). Despite its relative stability, personality is malleable and partially changes across the lifespan (26). These continuous developmental processes highlight the complexity of studying psychological traits during adolescence. Despite growing interest in the role of personality factors in youth player development, their specific role in elite youth football, particularly from a biopsychosocial perspective, remains understudied (29, 30). Based on Cloninger's biopsychosocial model, this study examines four personality dimensions - Novelty Seeking, Harm Avoidance, Persistence, and Self-Directedness—that have been linked to psychological mechanisms relevant to athletic development and injury risk in elite youth football (2, 5, 24). Specifically, Novelty Seeking and Harm Avoidance reflect motivational and emotional reactivity, which may influence an athlete's stress tolerance, risk behavior, and decision-making under pressure. In contrast, Persistence and Self-Directedness are considered indicators of self-regulation and goal-oriented behavior-traits essential for maintaining consistent training, overcoming setbacks, and achieving long-term performance goals (23, 31). Previous studies have shown that psychological characteristics such as intrinsic motivation, achievement orientation, and low anxiety are positively associated with athletic development and future success (2, 24, 32). According to our research, evidence on the association between personality dimensions and short-term physical performance remains scarce, particularly among elite adolescent athletes (5, 33). Recent reviews and meta-analyses indicate that traits such as conscientiousness, emotional stability, and extraversion are modestly but consistently associated with performance outcomes across different sports (13, 16, 34). Addressing this gap may provide novel insights into how temperament and character interact with motor development, talent progression, and injury risk in adolescent elite football players.

Beyond technical and tactical skills, physical performance in football is often assessed through standardized measures such as sprinting and jumping ability, which are considered key indicators of speed and explosive strength (35–37). While these sport motor tests capture important aspects of athletic capacity, their relationship with personality remains debated (1, 33). Previous studies have reported small and inconsistent associations between traits like conscientiousness, extraversion, and openness and physical performance indicators such as sprint capacity or heading ability (33, 38). However, investigating the adolescence as a critical period of physical and psychological development can provide new insights into talent development, training individualization, and long-term performance trajectories (39, 40).

In this regard, the Five-Factor Model (20) has been widely used, but it was originally developed for adults and only later adapted for adolescents. Youth-oriented versions such as the NEO-Personality Inventory-3 (NEO-PI-3) and NEO Five-Factor Inventory (NEO-FFI) have been validated in adolescent populations (40–43), yet evidence shows that personality traits display lower stability during adolescence, limiting their predictive power for developmental outcomes. By contrast, the Junior Temperament and Character Inventory (JTCI 12–18) was specifically designed and psychometrically validated for youth between 12 and 18 years, allowing reliable measurement of temperament and character during this developmental phase (28, 44).

As mentioned above, physical measures such as sprint and jump performance provide standardized and reliable indicators of speed and explosive strength, they capture only a limited part of football-specific performance. More comprehensive approaches also consider objective data from match play (e.g., tracking metrics, performance indices) and subjective evaluations such as coach or expert ratings, which may better reflect an athlete's overall effectiveness on the pitch (37, 45). From this perspective, personality may play a role not only in isolated motor tasks but also in broader aspects of game intelligence, tactical adaptability, and resilience, which are central to long-term success in elite football.

Football performance can be conceptualized broadly, encompassing technical, tactical, physical, and psychological components assessed through both objective match data and subjective ratings (37, 45). Within this broader framework, we focused on sprinting ability (30 m sprint) and jumping performance (heading jump) as two widely recognized indicators of speed and explosive strength (35, 36). These motor actions frequently precede goals, assists, and successful duels and are critical to in-game performance (37). Sprint capacity and jumping performance usually improves with age due to increased muscle mass and improved neuromuscular coordination. These factors are closely linked to biological maturation processes, such as peak height velocity and bone age (46, 47). Though context-dependent, sprint and jump tests offer standardized, valid, and reliable evaluations of physical preparedness outside match play (48, 49). The selection of these performance tests is supported by developmental considerations. Adolescence is a critical phase during which physical and psychological traits change (39, 40). Sprint and jump performance are widely used indicators of speed and explosive strength in youth football, yet previous studies report inconsistent associations between personality traits and isolated motor tasks (50, 51). This highlights the need to consider psychological factors within a broader developmental and behavioral context rather than focusing solely on isolated physical outputs (13).

Football has a higher injury incidence compared to many other sports and also a relatively high injury rate, with four to seven injuries per 1,000 playing hours (52). Furthermore, the injury rate during training for youth players is 2.3–4.9 times higher than that for adult players (53). The most common injuries affect the lower extremities, particularly the knee, ankle, and thigh, often in the form of sprains or muscle strains (54–56). Injury risks can be categorized into intrinsic factors (e.g., previous injuries, age, match frequency, psychological characteristics) and extrinsic factors (e.g., training intensity, field conditions), with intrinsic factors playing a particularly important role (55, 57). Like motor performance, research on the association between personality and injury susceptibility remains inconclusive. Recent studies have further emphasized the role of psychological and personality-related factors in injury susceptibility (19, 58). Junge and colleagues (59) linked high competition anxiety to increased injury risk, while Fuller (60) and Junge (24) found that athletes prone to injuries often exhibit higher risk-taking behavior and reduced caution. More recent studies also point to perfectionism, trait anxiety, and neuroticism as relevant risk factors, suggesting that maladaptive personality traits may influence stress responses, coping strategies, and ultimately injury susceptibility (16, 61). No conclusive personality profile linked to injury susceptibility has been identified.

While correlations between personality and performance are well-documented in academic and professional contexts, their direct role in elite football remains to be further elucidated (14). Personality dimensions can play an important role in elite youth football in two ways: First, they can aid in the selection of promising young players by providing insight into behavioral tendencies (14). Second, understanding personality dimensions enables more targeted talent development by reinforcing individual strengths and addressing areas for improvement (62). A deeper understanding of the relationship between personality and athletic performance may not only refine scouting methods but also facilitate psychological interventions in training programs, optimizing young athletes’ development (32, 63).

This study aims to address these research gaps by (1) describing the distribution of personality dimensions according to Cloninger's biopsychosocial model in a large sample of German elite youth football players, and (2) providing exploratory analyses of potential associations between these personality profiles and key motor performance parameters (sprint and jump ability) as well as injury frequency.

## Materials and methods

### Study design and population

This prospective, observational, cross-sectional study included football players from two German elite youth academies, both affiliated with professional men's teams competing in the first and second national divisions. Due to the mandated gender segregation in youth football (11), the sample exclusively comprised male participants. Data collection took place during the annual Pre-Competition Medical Assessments (PCMAs) conducted in the pre-season period of June to August 2023. This cross-sectional analysis represents the initial phase of a larger prospective project designed to longitudinally investigate the psychological, physical, and injury-related development of elite youth football players. Insights gained from the first data collection are intended to inform the refinement of hypotheses and methods in subsequent follow-up assessments. In both academies, the training load was progressively structured: U12 players trained three times per week, U13-U15 players trained four times per week, and U16-U23 players trained five times per week. Matches, either friendly or competitive, were typically scheduled on weekends throughout the season. The youth football schedule in Germany closely follows the professional league schedule, with a first half of the season from August to December and a second half from February to May.

Inclusion criteria required players to be aged 11–23 years and to have been enrolled in one of the clubs for at least one year. Exclusion criteria included withdrawal of informed consent by the player, parent, or legal guardian. To ensure psychometric validity, personality was only assessed in players within the validated 12–18 age range of the JTCI. Consequently, data from players younger than 12 or older than 18 were excluded from the personality analyses. Verbal consent was obtained from all players, while written informed consent was secured from at least one parent or other legal guardian before participation. Of the initial 228 participants, 35 were excluded due to missing data in the questionnaire (*n* = 17; e.g., anthropometric measures or injury data) and/or in sport-specific performance tests (*n* = 28), with some participants meeting both exclusion criteria. Consequently, the final analysis was conducted with a sample of *n* = 193 participants aged between 11 and 18 years (MW = 14.7; SD = 1.9; Table 1). The study adhered to the latest version of the Helsinki Declaration and was approved by the local ethics committee (Ulm University, No. 371/23).

**Table 1:** Participant characteristics, performance tests and lower limb injury incidence of the elite youth football players.

Age	Total players	Body mass	Body height	BMI	SMM/body height	PBF	Sprint 30 m	Heading jump	Incidence/1,000 h	Playing level
[Years]	[*n*]	[kg]	[cm]	[kg/m^2^]	[kg/m^2^]	[%]	[sec]	[cm]	[*n*]	[National division]
11	4	38.2 (6.7)	151.6 (12.2)	12.5 (1.3)	7.8 (0.1)	12.2 (4.4)	5.0 (0.3)	39.6 (2.2)	0.0	6th
12	27	43.0 (6.5)	156.2 (7.1)	13.8 (1.8)	8.4 (0.9)	9.5 (5.9)	4.9 (0.3)	39.2 (4.8)	1.8	4th–5th
13	27	49.6 (8.4)	165.4 (8.1)	14.9 (2.0)	8.8 (1.1)	10.5 (4.5)	4.7 (0.2)	46.9 (5.1)	0.9	2nd–3rd
14	32	58.8 (10.7)	171.1 (7.3)	17.1 (2.6)	10.0 (1.2)	8.7 (2.6)	4.4 (0.2)	50.7 (6.0)	0.7	2nd–3rd
15	32	64.2 (7.4)	176.1 (6.0)	18.2 (1.7)	10.5 (0.8)	9.6 (3.6)	4.4 (0.2)	50.7 (5.5)	1.0	2nd–3rd
16	38	68.1 (6.7)	178.0 (5.7)	19.1 (1.6)	11.0 (0.7)	9.7 (4.0)	4.2 (0.2)	53.5 (5.3)	0.8	1st–2nd
17	18	69.1 (6.3)	178.6 (5.9)	19.3 (1.4)	11.1 (0.9)	9.9 (1.6)	4.2 (0.1)	57.2 (9.0)	0.8	1st–2nd
18	15	74.1 (8.8)	179.4 (6.5)	20.6 (1.9)	11.7 (0.9)	9.9 (2.6)	4.2 (0.1)	55.4 (4.6)	1.6	1st–2nd

BMI, body mass index; SMM, skeletal muscle mass; PBF, percent body fat. Data except for age, total players, incidence and playing level are presented as mean and standard deviation.

### Data collection

The annual PCMA was conducted each summer before the start of the regular football season by the medical staff of the Division of Sports and Rehabilitation Medicine at the University Hospital Ulm, Germany. Once eligibility for study was confirmed, participants underwent additional assessments of anthropometry, performance tests and injury status.

### Anthropometrics

Body composition was analyzed using bioelectrical impedance technology (InBody 770, Biospace Korea, Seoul, Korea) to measure body mass [kg], fat-free mass (FFM) [kg/m^2^], and body fat percentage (BFA) [%]. The InBody intraclass correlation coefficients (ICC) for the measurement of body mass, fat-free mass (FFM), and body fat percentage (BFA) are at least 0.98, with a standard error of measurement (SEM) of less than 1% for BFA and less than 1 kg for FFM (64). Body height was measured without shoes using a portable stadiometer with 0.1 cm precision (seca213, Hamburg, Germany).

### Personality assessment

The Junior Temperament and Character Inventory (JTCI) 12–18 (44) is a validated self-report instrument with 103 items designed to assess personality dimensions in adolescents aged 12–18 years, based on the biopsychosocial model by Cloninger and colleagues (23, 28). The JTCI 12–18 comprises four temperament dimensions (Novelty Seeking, Harm Avoidance, Reward Dependence, and Persistence) and three-character dimensions (Self-Directedness, Cooperativeness, and Self-Transcendence), providing a comprehensive framework for personality assessment in adolescents. Its psychometric properties and applicability in adolescent populations have been supported by empirical research (44).

### Sprint performance assessment

Sprint performance was evaluated using a 30 m sprint test, a widely used method for assessing linear speed in football (35, 65). Time measurement was conducted using the WITTY timing system (WITTY Microgate, 2024), which features a high-precision photocell system with an accuracy exceeding 0.001 s. The system demonstrates high test-retest with an ICC of 0.96–0.99 (66). To ensure standardized test conditions, sprints were performed on artificial turf under comparable weather conditions. Players started in a standing position with a staggered stance and initiating the sprint at their discretion. The final sprint time was calculated as the mean of the three dimensions (35), which was then normalized to body height squared to account for anthropometric influence in further analyses.

### Jump performance assessment

Jump performance was assessed using a football-specific heading jump. A piezoelectric 1D force plate (Quattro Jump, Type 9290DD, Kistler, Winterthur, Switzerland) was used for measurement, with confirmed high reliability (ICC = 0.92–0.98) (50). Players were instructed to stand at the upper right corner of the force plate with both feet placed equally for symmetrical weight distribution. The jump was initiated by a two-step diagonal approach, followed by a maximal vertical jump from the center of the plate. A suspended ball served as a visual target but was not meant to be touched. Players were allowed to use their arms for momentum, ensuring a realistic heading movement (67, 68). Jump height was recorded in centimeters, and each player performed three dimensions. This test is considered highly reliable, with an ICC of 0.98 (69). The mean jump height from the three dimensions was converted to meters and normalized to body height squared to account for anthropometric effects. The resulting value was used for further analyses.

### Injury surveillance

The questionnaire followed the Fédération Internationale de Football Association (FIFA) Medical Assessment and Research Center (F-MARC) consensus on definitions and data collection procedures for football injury research. It incorporated translated versions of the standardized forms developed by Fuller et al. (25). The study recorded all traumatic musculoskeletal injuries, with a specific focus on lower limb regions (hip, thigh, knee, ankle, and foot) and injury types, including joint/ligament injuries, muscle/tendon injuries, and fractures. In accordance with F-MARC guidelines, an injury was defined as any physical complaint sustained by a player that results from a football match or football training, irrespective of the need for medical attention or time loss.

Participants completed the questionnaire in collaboration with their parents, reporting injuries sustained throughout their football careers and during the previous competitive season. All completed questionnaires were reviewed by medical staff, including sports scientists and physicians from the Division of Sports and Rehabilitation Medicine at the University Hospital Ulm. Only the total number of injuries was relevant for the present study. Therefore, all reported injuries were summed.

### Statistical analysis

All statistical analyses were conducted using RStudio (version 2022.07.2 + 576, RStudio Team, 2020), with a significance level of *p* ≤ .05. The JTCI 12–18 was administered via paper-pencil, requiring manual data entry. A plausibility check ensured data accuracy, revealing no irregularities. We transformed personality dimensions into percentile ranks based on the normative data provided in the JTCI manual (44) and calculated frequency distributions for all seven dimensions. For further analyses, we focused on Novelty Seeking, Harm Avoidance, Persistence, and Self-Directedness because they have been theoretically and empirically linked to key psychological factors relevant in elite sports contexts. Specifically, Novelty Seeking and Harm Avoidance reflect emotional reactivity and motivational tendencies, while Persistence and Self-Directedness are associated with goal-directed behavior and self-regulation-capacities that are critical for performance development and injury prevention. Our selection was informed by prior research emphasizing the predictive value of these traits for athletic motivation, coping with stress, and long-term engagement in youth football (2, 5, 32).

The Shapiro–Wilk test assessed normality, indicating significant deviations in most distributions (70). Consequently, Spearman's rank correlation was used (71), with Holm´s correction for multiple comparisons (72). Correlations with age were calculated, followed by partial correlations controlling age-related effects. Moderation hypotheses were tested using multiple linear regression with interaction terms. Assumptions of linearity (scatterplots), multicollinearity (correlation matrices, VIF values; *r* > 0.8, VIF < 10 (73), and homoscedasticity (residual plots) were checked. HC3 robust standard errors were applied when necessary (74), and Cook's distance confirmed the absence of influential outliers. Q-Q plots were used to validate the normality of residuals. Three outcome variables were analyzed: 30 m sprint time, heading jump height, and injury frequency. Age was included as both a moderator and a main effect in all models. Separate regression models were calculated for each personality dimension: Novelty Seeking, Harm Avoidance, Persistence, and Self-Directedness, as predictors. Only models that met statistical assumptions were included. Since injury frequency ranged from 0 to 5, a dichotomous variable (0 = no injury, 1 = ≥1 injury) was created, and logistic regression was used. In all analyses, significant moderation effects were compared to non-interaction models to assess model fit.

## Results

**Figure 1:**
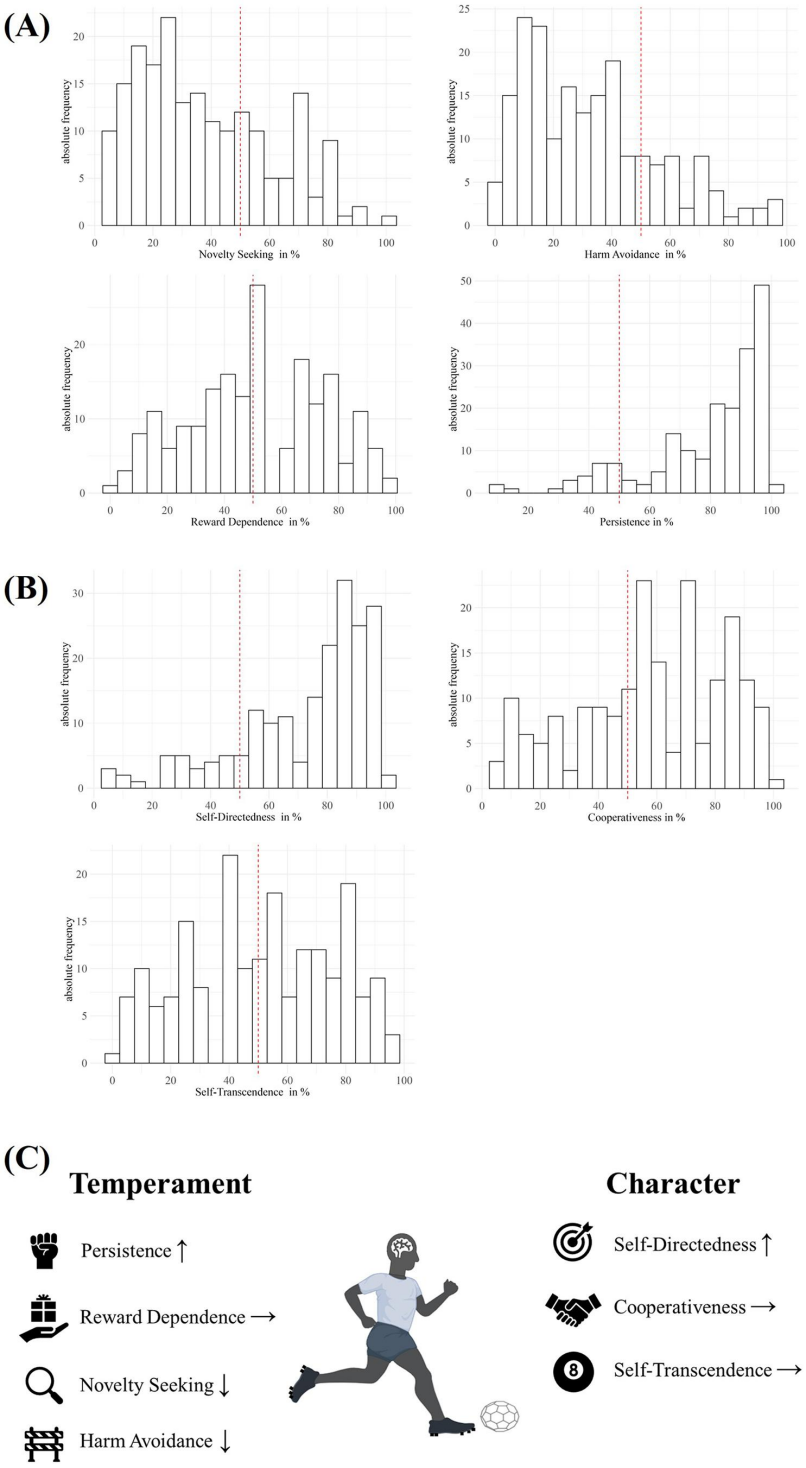
Distribution of Cloninger's personality dimensions in elite youth football players. The personality scale scores were converted to percentile ranks based on a normative sample. Frequency histograms for the seven assessed personality dimensions are shown, grouped into **(A)** the four temperament dimensions and **(B)** the three character dimensions. **(C)** summarizes distributional characteristics of all seven dimensions from **(A)** and **(B)** Arrows indicate the skewness of the distribution of the percentile ranks of each dimension. The *x*-axis in **(A)** and **(B)** represents percentile ranks (%), the *y*-axis the number of players. Red dashed vertical lines indicate the median of each distribution. In **(C)**, upward pointing arrows indicate a left-skewed distribution, i.e., a dominance of high percentile ranks. Downward pointing arrows indicate a right-skewed distribution, i.e., a dominance of lower percentile ranks. A horizontal arrow pointing to the right indicates a distribution with little skewness, i.e., dominance of intermediate percentile ranks or an even distribution.

The internal consistency of the JTCI questionnaire was acceptable to good (Cronbach's *α* > 0.66) and is presented along with the mean values and standard deviations for each personality dimension in Supplementary Table S2. The personality scale scores of elite youth football players were converted to percentile ranks based on the normative sample. Figure 1 presents frequency histograms for the seven assessed personality dimensions, grouped into temperament (A) and character dimensions (B).

### Distribution of the personality dimensions

The distribution of temperament dimensions (Figure 1A) showed the following characteristics: Novelty Seeking had a mean of 37.35 (SD = 54.00), a median of 34, and a positively skewed distribution (skewness = 0.55), with the mode at percentiles 20%–25%. Harm Avoidance had a mean of 32.56 (SD = 46.00), a median of 31, and a positive skewness of 0.80, with a mode at percentiles 10%–15%. Reward Dependence was approximately normally distributed (skewness = −0.05) with a mean of 51.18 (SD = 73.00), a median of 50, and the mode at percentiles 50%–55%. Persistence had a mean of 79.79 (SD = 95.00), a median of 86, and was strongly left-skewed (skewness = −1.38), with 50% of values between percentiles 69 and 95, and a mode at percentiles 95%–100%. The character dimension Self-Directedness had the highest values (MW = 73.35, SD = 90.00, median = 82) with a left-skewed distribution (skewness = −1.17) and a mode at percentiles 85%–90%. Cooperativeness (MW = 58.21, SD = 79.00, median = 62) showed a slight left skew (skewness = −0.39). Self-Transcendence (MW = 51.90, SD = 73.00, median = 50) exhibited a broad, multimodal distribution with minor skewness (−0.18) and a mode at percentiles 40%–45% (Figure 1B). Figure 1C summarizes all seven dimensions using arrows to indicate the orientation of each dimension according to its percentile rank.

### Correlation analyses

In further analyses, Novelty Seeking, Harm Avoidance, Persistence, and Self-Directedness were identified as key personality dimensions. Their associations with performance parameters were examined, while controlling for age (Table 2). Significant correlations were observed only between age, 30 m sprint time, and injury frequency. No significant correlations were found between the personality dimensions Novelty Seeking, Harm Avoidance or Self-Directedness and injury frequency, 30 m sprint performance, or heading jump height. In contrast, Persistence showed a significant negative correlation with heading jump performance, but not with injury frequency or sprint performance (Table 3).

**Table 2:** Spearman rank correlation coefficients of the correlations between age and the relevant personality dimensions and the sport motor performance parameters.

Statistic	Novelty seeking	Harm avoidance	Persistence	Self-Directedness	Sprint 30 m	Heading jump	Injury frequency
Age *r_SP_*	.04	.02	−.13	.003	−.77***	.13	.22**
*n*	193	193	193	193	193	193	193

Significant correlations are in bold. **p < .01; ***p < .001. All values are standardized. Rsp, Spearman rank correlation coefficient; n, number of subjects.

**Table 3:** Spearman's partial rank correlation coefficients between relevant personality dimensions and motor performance parameters, controlling for age.

Personality dimensions	Statistic	Sprint 30 m	Heading jump	Injury frequency
Novelty seeking	*r_SP_*	.024	.064	.061
Harm avoidance	*r_SP_*	.020	.049	−.020
Persistence	*r_SP_*	−.012	−.200*	.016
Self-Directedness	*r_SP_*	−.040	-.070	−.037

Significant correlations are highlighted. *p < .05. All scores are standardized. Corrected for multiple testing using the Holm procedure. r_SP_ = Spearman rank correlation coefficient. Number of subjects *n* = 193.

### Moderation analyses

Despite the lack of significant associations in the preliminary correlation analyses, moderation models were conducted to examine whether age moderates the relationship between personality dimensions and athletic performance.

### 30 m sprint

All four regression models predicting 30 m sprint performance yielded statistically significant overall effects, with an adjusted coefficient of determination (*R*^2^) of 0.59 for the models including Novelty Seeking, Harm Avoidance, and Self-Directedness, and 0.60 for the model including Persistence. In each model, age emerged as a statistically significant predictor (all *β* = −0.24, all *p* < .001), indicating that increased age was associated with improved sprint performance (i.e., shorter sprint times).

No statistically significant interaction effects were found between age and any of the personality dimensions (all *p* > .05). In the model including Persistence, a marginally significant interaction was observed prior to the application of robust standard errors (*β* = −0.03, *p* = .049). However, after correction for heteroscedasticity, the interaction effect no longer reached the conventional threshold for statistical significance (*β* = −0.03, *p* = .079).

### Heading jump

Moderation analyses were also conducted to examine the relationship between personality dimensions and heading jump performance. None of the four models yielded statistically significant overall effects. The only statistically significant main effect was found in the model including Persistence (*β* = −0.003, *p* = .008), indicating a negative relationship between Persistence and jump performance. However, no statistically significant interaction effects between age and any of the personality dimensions were observed in any of the models.

### Injury frequency

In all four models, age was the only variable that significantly predicted injury occurrence (*p* = .001). The odds ratio for age was consistently 1.70 [95% confidence interval (1.24, 2.34)], suggesting a higher probability of injury with increasing age. No significant interaction effects between personality dimensions and age were identified in any of the logistic regression models. Across all analyses, age consistently emerged as a significant predictor of 30 m sprint performance and injury frequency, but not of heading jump performance. No statistically significant interaction effects between age and personality dimensions were observed in any of the models. An overview of all regression coefficients is presented in Table 4.

**Table 4:** Logistic moderation analyses examining the relationship between personality dimensions and injury occurrence, with age included as a moderating variable.

30 m-sprint	Personality dimension				Age				Personality dimension*age			
	β	SE	t	*p*	β	SE	t	*p*	β	SE	t	*p*
NV	−0.01	0.02	−0.52	.61	−0.24	0.02	−13.64	<.001	−0.01	0.02	−0.39	.70
SV	0.01	0.01	0.57	.57	−0.24	0.02	−13.51	<.001	0.01	0.02	0.48	.63
BV	0.003	0.01	0.23	.82	−0.24	0.02	−13.68	<.001	−0.03	0.01	−1.77	.08
SL	−0.004	0.02	−0.28	.78	−0.24	0.02	−13.58	<.001	−0.01	0.02	−0.59	.56
Heading jump												
NV	0.002	0.002	1.20	.23	0.003	0.001	1.46	.16	−0.002	0.002	−1.3	.20
SV	0.000	0.002	0.47	.64	0.003	0.002	1.60	.11	−0.001	0.002	−0.61	.54
BV	−0.003	0.001	−2.67	.008	0.003	0.002	1.40	.17	0.000	0.001	0.22	.82
SL	−0.002	0.002	−1.00	.32	0.003	0.002	1.60	.11	0.002	0.002	1.07	.29
Injury frequency	β	z	p	OR; 95%-CI	β	z	p	OR; 95%-CI	β	z	p	OR; 95%-CI
NV	0.19	1.23	.22	1.21 [0.89, 1.66]	0.52	3.20	<.001	1.70; [1.24, 2.34]	−0.06	−0.37	.71	0.94; [0.68, 1.31]
SV	−0.09	−0.58	.56	0.91 [0.66, 1.24]	0.52	3.20	<.001	1.70; [1.24, 2.34]	0.16	0.93	.35	1.17; [0.84, 1.64]
BV	0.05	0.31	.75	1.05 [0.77, 1.46]	0.52	3.20	<.001	1.70; [1.24, 2.34]	0.01	0.043	.97	1.01; [0.75, 1.34]
SL	0.10	0.61	.54	1.11 [0.81, 1.54]	0.52	3.20	<.001	1.70; [1.24,2.34]	−0.17	−0.97	.33	0.84; [0.59, 1.19]

Significant *p*-values are highlighted. β represents standardized regression coefficients. OR, odds ratio; 95% CI, 95% confidence Interval. NV, novelty seeking; SV, harm avoidance; BV, persistence; SL, Self-Directedness. Number of subjects *n* = 193.

## Discussion

This prospective, observational, cross-sectional study included 193 male football players from two German elite youth academies aged between 11 and 18 years. The aim of the present study was to explore the relationship between the personality dimensions of the biopsychosocial personality model and the sport-specific performance of elite youth football players, as well as to examine the moderating effect of age on these relationships. Our main finding was that elite youth football players exhibited particularly high levels of Persistence and Self-Directedness, while dimensions such as Novelty Seeking and Harm Avoidance were comparatively low. Other personality dimensions, including Reward Dependence, Cooperativeness, and Self-Transcendence, showed more balanced distributions across the sample. No significant associations were found between personality dimensions and motor performance, indicating that personality may not be directly related to physical performance in a cross-sectional context.

### Personality dimensions in elite youth football players

Our results showed that elite youth football players were characterized by high levels of Persistence and Self-Directedness and low levels of Novelty Seeking and Harm Avoidance. To our knowledge, this is the first study to systematically describe the temperament and character dimensions of elite youth football players in Germany based on Cloninger's biopsychosocial model. The athletes’ personality profiles were characterized by high Persistence and Self-Directedness and low levels of Novelty Seeking and Harm Avoidance. This pattern of high Persistence and low Harm Avoidance is consistent with international meta-analytic findings showing that successful athletes typically display higher levels of self-discipline and lower emotional reactivity compared to non-elite performers (13). These traits are also crucial for managing stress and achieving long-term goals in competitive sports (23, 28, 32, 75). The observed homogeneity in certain traits may suggest implicit selection preferences in elite youth football environments, favoring players with high self-regulation and low emotional reactivity. While this may promote consistency and discipline, it could also diminish psychological diversity within teams. For example, players with higher novelty seeking may contribute creative and unpredictable elements to matches, which could be advantageous with the right support (6). The cross-sectional correlational design of our study and these descriptive findings do not allow for causal conclusions, but they may serve as a basis for future research into psychological factors in player development. Comparing elite players to non-elite or professional samples could help determine if this personality profile is specific to the elite pathway or if it emerges over time. In summary, the personality profiles observed in our sample suggest that certain temperament and character traits may be overrepresented in elite youth football, possibly reflecting selection effects or environmental adaptations within high-performance settings.

### Relationships between personality dimensions and sport-specific performance parameters in elite youth football players

Our results showed no significant associations between the four investigated personality dimensions—Novelty Seeking, Harm Avoidance, Persistence, and Self-Directedness—and sport-specific performance measures (30 m sprint and heading jump). The only exception was a moderate negative correlation between Persistence and heading jump height, contrary to our initial hypothesis. This unexpected finding may reflect the nature of Persistence as a trait associated with perfectionism and methodical behavior (28). This trait could potentially impair the explosive qualities required for maximal jump performance (76). The absence of broader associations indicates that personality traits may have limited relevance for explaining the current short-term motor performance of this highly selected group of elite youth players.

The current findings should be viewed against the backdrop of prior research, which has consistently reported small and inconsistent associations between personality traits and motor performance (1, 33, 77, 78). As outlined in the introduction, variations in study design, outcome measures [e.g., heading frequency vs. jumping ability (38)], and sample characteristics may account for these discrepancies. In line with previous work, our study supports the notion that while psychological traits may contribute to athletic behavior, their predictive value for isolated motor outcomes remains limited when compared to physical and maturational factors.

Moreover, performance tests like the 30 m sprint and heading jump capture a snapshot of physical ability at a given point in time. These outcomes are likely more influenced by physiological and biomechanical factors, such as strength, flexibility, and VO_2_max, than by stable psychological traits (79). In contrast, personality may exert its influence over the long term. While sprinting and jumping ability can improve with training, the ability to train consistently and overcome setbacks may reflect underlying personality traits. Rather than directly predicting motor performance, personality may shape developmental pathways that distinguish good athletes from elite ones (80). Therefore, personality may be more relevant to complex performance aspects—such as game intelligence, tactical adaptability, and psychological resilience—than to basic motor tasks.

Studies suggest that traits such as motivation, self-regulation, and low anxiety are linked to future athletic development (2, 5, 24, 32), supporting this perspective. Aidman (80) and others (33, 62, 81) emphasize the role of personality in predicting long-term success when other factors remain stable. Consistent with this idea, Höner and Feichtinger (5) found that motivational and self-regulatory traits were unrelated to current performance measures but predictive of future achievement. Regarding injury risk, our findings align with previous literature indicating that no specific personality profile reliably predicts injury susceptibility (24). Rather, state-like psychological stress may temporarily impair neuromuscular function and increase injury risk (82). Although the cross-sectional design does not allow causal inference, previous research suggests that personality traits may influence performance and injury risk indirectly through behavioral mediators. For example, higher Self-Directedness is associated with better adherence to training and recovery routines, which may reduce fatigue-related injury mechanisms (77). Conversely, higher Novelty Seeking has been linked to impulsive decision-making and reduced error monitoring under pressure, potentially increasing exposure to high-risk movement patterns (59). These behavioral tendencies offer a plausible pathway through which personality may contribute to athletic development and resilience over time. In summary, within the framework of Cloninger's biopsychosocial model, our findings suggest that personality traits may not strongly predict immediate physical performance in elite youth football. However, they may offer valuable insights into players’ long-term developmental trajectories, explaining why some athletes reach elite levels while others do not.

### Age as a moderator of the relationship between relevant personality dimensions and sport-specific performance parameters in elite youth football players

Our analyses showed that age was significantly associated with sprint performance and injury frequency but not with heading jump height or any of the investigated personality dimensions. Sprint ability improved with age; however older players also exhibited a higher injury frequency. In contrast, Novelty Seeking, Harm Avoidance, Persistence, and Self-Directedness showed no significant correlation with age. These findings are consistent with theoretical assumptions and previous research. The stability of temperament dimensions across age aligns with Cloninger's model (23), while the absence of age effects on Self-Directedness corresponds with findings by Lenz et al. (63), who reported little age-related variance in conscientiousness within performance-oriented samples. The negative correlation between age and sprint times confirms previous studies showing improvements in sprint capacity during adolescence (79). The increase in injury frequency with age is also well-documented and likely results from cumulative physical load and a growing history of prior injuries (83–86).

Regarding potential moderation effects, age did not moderate the relationship between Novelty Seeking, Harm Avoidance, or Self-Directedness and sprint performance. However, an age-dependent pattern emerged for Persistence: older players showed a weak positive trend between Persistence and sprint ability, whereas younger players displayed a slight negative association. Although not statistically robust, this pattern may reflect the growing relevance of discipline and effort with increasing age (28). Across all regression models, age was the strongest predictor of sprint performance, explaining approximately 59% of the variance. This supports previous findings that peak performance occurs in late adolescence due to physical maturation and gains in muscle mass and strength (79, 87). No significant interactions between age and personality were found in relation to heading jump height. This likely reflects the influence of neuromuscular coordination and explosive strength, which are more effectively enhanced through training than shaped by personality traits (76). Age was also a significant predictor of injury frequency, with each standard deviation increase corresponding to a 70% higher risk of injury. These findings support those linking older age with a higher risk of injury due to accumulated load and injury history (54, 85, 88–90). In summary, age plays a direct and significant role in predicting sprint capacity and injury frequency. However, in this cross-sectional analysis, personality dimensions showed no predictive value for these outcomes, suggesting that they may be more relevant for long-term development than short-term physical performance markers (91, 92).

From a practical standpoint, the results may provide coaches and practitioners with useful starting points for individualizing training and development strategies. Players with high Persistence and Self-Directedness are typically characterized by perseverance and goal-oriented self-regulation, which could be fostered by autonomy-supportive coaching and structured long-term development planning (23, 28, 32). Conversely, players with higher levels of Harm Avoidance may benefit from emotionally supportive environments and coaching strategies that reduce anxiety and enhance coping under pressure (6, 75). Finally, players with high levels of Novelty Seeking may need support to manage impulsivity and to improve commitment to training rules, goal maintenance and goal-directed action over a longer period. Understanding these personality profiles may help coaches optimize motivation, communication, and role assignments, ultimately enhancing athletic performance and psychological resilience in young athletes. This aligns with recent research that calls for a more personalized approach to athlete development that integrates psychological characteristics into long-term talent identification systems (8, 63).

### Limitations

As a cross-sectional study, causal and developmental conclusions cannot be drawn. Longitudinal designs are necessary for exploring how personality influences long-term athletic performance trajectories (2, 80). Another important aspect concerns the exclusive use of self-reports, which may be affected by subjective bias. While standardized instruments like the JTCI provide valid psychometric assessments, self-perceptions can be influenced by factors such as social desirability or limited self-awareness. The inclusion of external ratings, particularly from coaches, could help to increase the objectivity and ecological validity of the data. Coaches are generally regarded as experts in evaluating players’ behavior and potential over time, and their assessments are increasingly recommended in psychological talent diagnostics (6, 8, 32).

In addition, this study focused exclusively on physical performance parameters such as sprint and jump performance, while other key indicators of football-specific success—such as technical and tactical skills, game intelligence, or actual match performance—were not assessed. Furthermore, the sample consisted exclusively of male players from elite youth academies, which may have led to a bias in the selection. Psychosocial influences, including support from parents, coaches, peers, and the broader social environment, were also not included, despite their recognized importance in youth player development (3). While associations between psychological traits and injury risk were explored, it is important to acknowledge the multifactorial nature of injuries. Psychological traits represent only one of many contributing factors, and causal interpretations are not possible due to the cross-sectional design. Moreover, the data did not allow a distinction between contact and non-contact injuries, limiting interpretation of injury mechanisms.

Future research should therefore adopt a more comprehensive approach that integrates psychological, physical, technical, and social factors within a longitudinal design to better capture the complexity of talent development in elite youth football. Comparative analyses involving non-elite youth and professional players may also help to identify developmental patterns and distinct personality profiles across performance levels. In addition, we acknowledge the conceptual limitations of isolated fitness tests and recommend that future studies combine personality assessments with match-specific performance metrics derived from tracking systems to enhance ecological validity and practical relevance.

## Conclusion

This study examined the relationship between personality traits, physical performance, and injury risk in 193 elite youth football players. The athletes showed high levels of Persistence and Self-Directedness and low levels of Novelty Seeking and Harm Avoidance-traits that may reflect functional characteristics of elite youth football environments, such as self-regulation, goal orientation, and emotional stability. Although personality was not associated with short-term sprint or jump performance, age was clearly the strongest predictor of sprint capacity and injury frequency. A weak age-dependent trend for Persistence further suggests developmental dynamics in the interplay between personality and physical performance.

The results emphasize the value of integrating personality assessments into individualized support strategies in youth player development. Traits such as Persistence, Self-Directedness, or Harm Avoidance can offer important insights into how athletes respond to training, pressure, and long-term goals, enabling more tailored and psychologically informed coaching. These findings underscore the need for longitudinal research to examine how personality influences athletic growth over time. We also emphasize that personality is malleable, especially during adolescence, and that personality traits in adolescent athletes may be limited in predicting future success in adulthood. This analysis marks the beginning of a long-term research project that will monitor the interplay of psychological and physical performance-related factors throughout the developmental trajectory of elite youth players.

## Data Availability

The original contributions presented in the study are included in the article/Supplementary Material, further inquiries can be directed to the corresponding author.

## Supplementary material

SUPPLEMENTARY TABLE S1 Descriptions and functional relevance of JTCI personality dimensions (23).

SUPPLEMENTARY TABLE S2 Internal consistency of the seven main scales of the JTCI 12–18. MW, mean; SD, standard deviation. Internal consistency is reported as Cronbach's α for each scale. Note: The sample size refers to all complete JTCI 12–18 responses.
